# “You Gotta Eat It to Survive”: A Mixed-Methods Study of the Perceived Food Environment and Food Choice Among Youth Experiencing Homelessness

**DOI:** 10.3390/nu18182963

**Published:** 2026-09-10

**Authors:** Sarah Beth Dunn, Stacy Vegas Lu, Natasha Slesnick, Julie Kennel, Irene E. Hatsu

**Affiliations:** 1 Department of Human Sciences, College of Education and Human Ecology, The Ohio State University, Columbus, OH 43210, USA; sarahbethdunn@outlook.com (S.B.D.); slesnick.5@osu.edu (N.S.); kennel.3@osu.edu (J.K.); 2 Department of Population, Family and Reproductive Health, Johns Hopkins Bloomberg School of Public Health, Baltimore, MD 21231, USA; svlu@memphis.edu; 3 OSU Extension, The Ohio State University, Columbus, OH 43210, USA

**Keywords:** youth, food insecurity, food choice, homelessness, food environment

## Abstract

**Background/Objectives**: This study aimed to examine how the perceived food environment influences food choice among youth experiencing homelessness. **Methods**: A convergent parallel mixed-methods design was used with 14 youth experiencing homelessness (aged 18–23 years; 64% Black/African American; 57% male) in Columbus, Ohio, United States. Data were collected through semi-structured interviews, surveys, and site observations between August 2022 and February 2023. Interviews and surveys assessed participants’ perceptions of their food environment and food choices. Interviews were audio-recorded, transcribed verbatim and analyzed by inductive thematic approaches to identify patterns, categories, and relationships. To quantify the physical food environment, observations were conducted at food acquisitions sites identified during interviews and analyzed descriptively. Qualitative and quantitative data were collected concurrently, analyzed separately, and subsequently integrated to examine areas of convergence and divergence. **Results**: Participants obtained food primarily from fast-food restaurants and a drop-in center, whereas grocery and corner stores were used less frequently. Although food was available through multiple sources within participants’ food environment, they were perceived as unaffordable, inaccessible, or unappetizing. Specifically, foods from grocery stores were perceived as unaffordable and inaccessible, while foods provided by the drop-in center and fast-food restaurants were viewed as unappetizing, unhealthy, and sometimes inaccessible. Observational data also showed variability in access to healthy food options across the various settings. Despite the availability of food within their environments, all participants experienced food insecurity. **Conclusions:** The food environment and food choice for youth experiencing homelessness is shaped not only by the mere availability of food but also its perceived affordability, accessibility, and desirability. Interventions need to extend beyond increasing food availability to ensure that available foods are affordable, accessible, and of high quality. Tailored education in nutrition, food safety, and food resource management may further enhance youths’ ability to identify, select, and prepare available foods in ways that increase their perceived desirability.

## 1. Introduction

An estimated 653,000 individuals experience homelessness in the United States, of which approximately 7.3% are youth aged 18–24 years who “lack a fixed, regular, and adequate nighttime residence” [1]. The transition from adolescence to adulthood is a critical period when youth have increased responsibility over themselves, including the expectation to obtain food independently [2,3]. Food obtainment demands an immense investment of time, energy, and resources, and unlike adult populations, youth experiencing homelessness (YEH) face unique challenges that shape how they access and consume food. This includes built environments that offer limited healthy food choices along with limited access to food preparation and storage facilities [4,5]. In addition, many YEH struggle with alcohol and substance misuse, which complicates the issue of feeding [6,7,8]. In this context, evidence shows that YEH experience food insecurity, or a “lack of consistent, dependable access to enough food for active, healthy living” [9,10,11,12,13]. Many youth experiencing homelessness endure prolonged involuntary food deprivation, while an even larger proportion report reductions in the quality and quantity of food consumed during episodes of homelessness [14]. To meet their food needs, YEH rely on a range of subsistence strategies, including panhandling, theft, drug sales, sex work and assistance from social networks [15]. However, reliance on these strategies may increase exposure to street victimization. Some studies have also documented the use of earned income or government food assistance benefits to purchase food [5,12]. Even so, having limited food resources, coupled with prolonged exposure to a heterogenous food environment, can contribute to micronutrient deficiencies, obesity, cardiometabolic risks, and poor mental health [16,17].

The food environment influences food choices, dietary patterns, and subsequent nutritional and health outcomes [18]. It has been defined to include the physical food environment (the availability, accessibility, and affordability of food within the built environment, including resources and infrastructure related to food acquisition, such as food retailers, transportation, and government assistance programs), the social food environment (norms, beliefs, and values related to food in one’s communities), and the person-centered food environment (a reflection of taste preferences, attitudes and experiences toward food), all of which influence each other in an overlapping manner [19]. An important aspect of this definition is that an individual’s perceptions of their food environment both shape and are shaped by their physical and social environments [19]. Further, an individual’s food choice is shaped by their perceptions of their food environment [20,21]. Among YEH, despite the evidence of high levels of food insecurity and exposure to poor physical food environments, limited research has examined how youth perceive and navigate these environments and how such experiences shape their food choices. In addition, due to their limited economic resources, YEH’s disproportionate exposure to unhealthy food environments makes them vulnerable to a greater burden of diet-related inequities [22]. These challenges are further compounded by the unique nutritional vulnerabilities associated with adolescence and young adulthood [23]. Therefore, understanding how youth perceive their food environment is integral for informing interventions aimed at both improving their food environment and nutritional well-being. Such insights can guide targeted interventions and inform food and nutrition security efforts in ways that are effective and responsive to the conditions in which they live [22]. This study, therefore, sought to examine how the perceived food environment influences food choice among youth experiencing homelessness based on the following aims: (1) explore how youth facing homelessness experience their food environment from a social and person-centered lens; (2) measure the perceptions youth facing homelessness have of their food environment; and (3) detail how youth’s social networks, personal beliefs, and physical environment influence food choice.

## 2. Materials and Methods

### 2.1. Study Design

This study used a convergent parallel mixed-methods design with qualitative prioritization (quan + QUAL) to examine how youth experiencing homelessness perceived their food environment and its influence on food choice. Interviews were conducted to collect qualitative data, based on a phenomenology with thematic analysis approach, while surveys and site observations were completed for the quantitative strand. The order in which the interviews and surveys were completed varied by individual participant preference. Interviews and surveys were completed between August and November 2022, and site observations were conducted between November 2022 and February 2023.

Qualitative and quantitative data were collected and analyzed simultaneously but separately; then, data were integrated and analyzed to examine points of consistency, discrepancy, and complexity. It was necessary to obtain and compare qualitative and quantitative data to gain a more comprehensive understanding of this understudied phenomenon. The qualitative data were prioritized due to the exploratory nature of the study and to ground the analyses in youth’s perspectives.

### 2.2. Study Site and Sample

Participants were recruited from a 24 h/seven-day-a-week drop-in center serving youth ages 14–24 years who were experiencing homelessness in Columbus, OH, USA. The drop-in center provided youth with access to communal facilities (kitchen, laundry) and resources (employment, health care). Food was available at the drop-in center and obtained via donations from various organizations and purchases from a local food bank. Youth were permitted to access this food and prepare meals at the drop-in center’s kitchen. Youth accessing the drop-in center were eligible for the study if they: (1) were experiencing homelessness as defined by the McKinney–Vento Act (2002): “lack[ing] a fixed, regular, and adequate nighttime residence.”; (2) were at least 18 years old; and (3) provided written informed consent. Recruitment occurred at the drop-in center via word-of-mouth and flyers and stopped when saturation was reached, defined as the point when no new concepts emerged from the interviews [24]. Participants received a $50 gift card after completing both the interview and survey. This study was approved by the Ohio State University Institutional Review Board (#2022B0208).

### 2.3. Interviews

Semi-structured individual interviews were employed in the qualitative arm of this study. Individual interviews were used because of the sensitive nature of the questions being explored and to provide participants with confidentiality to freely describe their experiences without concern about potential repercussions within their community. The interview guide was developed based on a review of the extant literature and guided by the Social Ecological Model, which contends that an individual’s beliefs and behaviors are influenced by a network of individual, relational, community, and societal factors [25]. Therefore, the interview guide included 13 open-ended questions about dietary patterns, food purchasing behaviors, taste preferences, food preparation skills, food accessibility, social norms about food, and food safety concerns (See Appendix A for Interview Guide). The interview questions were primarily adapted from previous research among other populations experiencing homelessness such as adults and families, and were selected to align with relevant constructs, theoretical frameworks, and study aims. They were further assessed for conceptual consistency and clarity before inclusion in the interview guide.

All interviews were conducted in person, by two trained interviewers (S.B.D. and a student assistant), in a private room at the drop-in center. Both interviewers were trained in focus group facilitation, and semi-structured interview techniques, and trauma-informed approaches to data collection. They also had an established rapport with the study population prior to data collection. Interviews continued until saturation was reached; lasted between 30 to 104 min and were audio-recorded, transcribed verbatim, and analyzed using MAXQDA 22 (VERBI Software, 2022, Berlin, Germany). Two researchers (S.B.D., and S.V.L.) inductively derived codes from the transcripts, evaluated the codes for patterns and natural categories, and investigated connections between the categories. Initial open coding was performed on two transcripts to develop a preliminary codebook, which was subsequently refined through coding and comparison of two additional transcripts. One researcher (S.B.D) coded all remaining transcripts, while the other (S.V.L.) independently coded every other transcript to enhance analytic rigor. The researchers met throughout analysis to discuss discrepancies, emerging themes, and alternative interpretations. To support reflexivity, one of the researchers (S.B.D.) maintained a bracketing journal documenting personal assumptions, biases, emotional responses, questions, and evolving interpretations of the data. Participant pseudonyms were created for the reporting of results.

### 2.4. Surveys

Interviewer-administered paper surveys were used to collect participant data about demographic characteristics (age, race/ethnicity, sex/gender, educational attainment, employment status, individual monthly income), places of shelter and the length of stay in such locations, receipt of Supplemental Nutrition Assistance Program (SNAP) benefits, food security status, and perceived food environment. Food security was measured using the six-item US Household Food Security Survey Module (HFSSM) [26]. Using standard scoring, participants were categorized into one of the following food security statuses, with higher scores indicative of poorer food security outcomes: high or marginal food security (0–1), low food security (2–4), or very low food security (5–6) [26]. Perceived food environment was assessed using the Perceived Nutrition Environment Measures Survey (NEMS-P) [27]. The NEMS-P was developed to measure three types of food environment: community nutrition environment (access to stores and restaurants within the neighborhood), consumer nutrition environment (availability, and cost of healthy food options, promotions, and nutrition information at stores and restaurants) and home food environment (availability and accessibility of healthy and unhealthy food at home), and includes questions on the importance of taste, nutrition, cost, convenience, and weight control when purchasing food at stores or restaurants. As NEMS-P is validated for use among housed populations with conventional definitions of “home”, limitations are noted (see Strengths and Limitations section) for its use in this context of housing instability. In the present study, home was operationalized as the location where youth slept at night, which for most participants was a shelter. All survey data were analyzed using univariate descriptive statistics in Stata 17.0 (StataCorp., 2021. College Station, TX, USA).

### 2.5. Site Observations

To quantify the physical food environment, site observations were conducted using the Nutrition Environment Measures Survey (NEMS) observational tools for restaurants, stores, and corner stores (NEMS-R, NEMS-S, and NEMS-CS), respectively. All these tools were scored using standardized scoring procedures that produce an overall score based on food availability, pricing, quality, facilitators, and/or barriers sub-scores. Briefly, points were assigned for availability when the food item was being sold (e.g., yes = 1 point, no = 0), for pricing when a heathier item is less expensive than a comparable high fat or high sugar alternative, for quality when more than 50% of the food items on the shelf are deemed acceptable, and for facilitators of healthy food choices when such supports are present. Conversely, points are deducted when healthier options were priced higher than less healthy alternatives or when barriers to healthy food choices were present. Higher total scores on the NEMS observational tools indicated greater availability and accessibility of healthy food at sites, whereas lower total scores indicated lower availability and accessibility of healthy food [28,29,30]. Healthy food was defined by the NEMS as entrees with up to 10% saturated fat and up to 800 calories (or 650 calories for burgers and sandwiches) [30] Data were collected about the type and location of food outlets; availability, placement, and promotion of healthful foods choices; and product information and pricing [31]. Sites for observation were identified based on participants’ interview responses; these were sites that youth identified as places where they routinely acquired food. Therefore, sites were excluded from observation if they were only mentioned by one participant and/or were not identified as places where food was acquired. There were instances where specific locations for some sites could not be determined based on participants’ interview responses. In such cases, the franchise located at the closest physical distance to the drop-in center was used for observation. This approach was based on evidence that walking was the primary mode of transportation for food access among youth and that many were recurrent users of the drop-in center. As such, franchise locations closer to the drop-in center were considered more likely to be patronized than those farther away. Similar to survey data, the observational data were analyzed using descriptive statistics in Stata 17.0 (StataCorp., 2021. College Station, TX, USA).

## 3. Results

A total of 14 participants completed the interviews and surveys. Participants were ages 18 to 23 years old (M ± SD: 20.57 ± 1.55), with a self-reported individual monthly income of $0 to $2600 (M ± SD: $935.17 ± 945.19). Most participants identified as non-Hispanic (93%), Black/African American (64%), and male (57%). Similarly, most participants completed at least high school education (57%) with 28% being employed part-time while the rest were students (36%), or unemployed or not working due to health reasons (36%). On average, participants reported experiencing homelessness for almost 9 months by the time of the study interview. Participants commonly reported spending the night in a shelter or mission; a car, abandoned building, or public indoor space; and/or an outside location (Table 1).

Four patterns reflecting youth’s perceptions of their food environment and its influence on food choice were determined: (1) Food is available in the youth’s environment but in a mixed landscape. (2) Available food is unappetizing to youth. (3) Appetizing food is unaffordable in the youth’s environment. (4) Appetizing food is inaccessible in the youth’s environment.

### 3.1. Food Is Available in the Youth’s Environment but in a Mixed Landscape

According to the NEMS observation measures, participants acquired food from fast-food restaurants, sit-down restaurants, grocery stores, and corner stores. Participants’ NEMS-P survey responses indicate they generally visited fast-food restaurants three times per week and sit-down restaurants once per week. Half of the participants reported shopping at grocery or corner stores once per month. Interview responses identified 27 food acquisition sites for observation, 26 of which were confirmed for assessment to include 22 fast-food and sit-down restaurants, three grocery stores, and one corner store. Across the 22 fast-food and sit-down restaurants, a total of 589 main entrees were identified.

In the interviews, participants reported additional places of food access including the drop-in center, home, food pantries, food banks, and soup kitchens. NEMS does not currently have tools to measure the food environment at drop-in centers or community establishments that serve free meals, so these locations could not be included in quantitative analyses in this study. Yet, the drop-in center was identified by all but one participant as an important place of food access. In fact, half of the participants stated they almost exclusively relied on the drop-in center to access food; most stating the drop-in center was the only place they had gotten food in the previous three months or since losing stable housing. Participants mentioned the availability of specific foods at the drop-in center including baked goods, yogurt, salad, chips, sandwiches, spaghetti, chicken, vegetables, fruit, and milk.

Most participants indicated on the NEMS-P that they had a stove, oven, refrigerator, freezer, and/or microwave present at the place where they usually slept at night. However, the places where participants usually slept at night did not always overlap with the places where they prepared, stored, or accessed food. In interviews, most participants named the drop-in center or a family member’s house as sites for food preparation and storage. For one participant, Khalil, having prior cooking skills enabled him to use the shared kitchen at the drop-in center to prepare food. However, for another participant, Jackson, having prior cooking skills helped him avoid using the kitchen at the drop-in center by instead using a homemade firepit outside his tent. Overall, participants described a range of cooking abilities, with the majority sharing that they primarily prepared premade, frozen, and/or prepackaged meals. As Jelani shared, “I cook cereal, toast, noodles, yeah. Cocoa, hot coffee. I don’t mean to be Gordon Ramsay and all, but… I do specialize in cereal.” Some youth mentioned adding additional ingredients and seasonings to make these foods more palatable.

### 3.2. Available Food Is Unappetizing to Youth

Although food was available, and accessible at the drop-in center and fast-food restaurants, every participant reported experiencing food insecurity. In fact, most participants (79%) were experiencing very low food security. Youth described their experiences of reduced food intake and hunger during the interviews, explaining that the food that they ate was often not the type of food they wanted to eat (referred to herein as appetizing or desirable food). As Andre explained, eating unappetizing foods could cause a “stomachache and makes you lightheaded. It makes me kind of irritable ‘cause I want something that I have a taste for, but I don’t really have it.”

**Figure 1:**
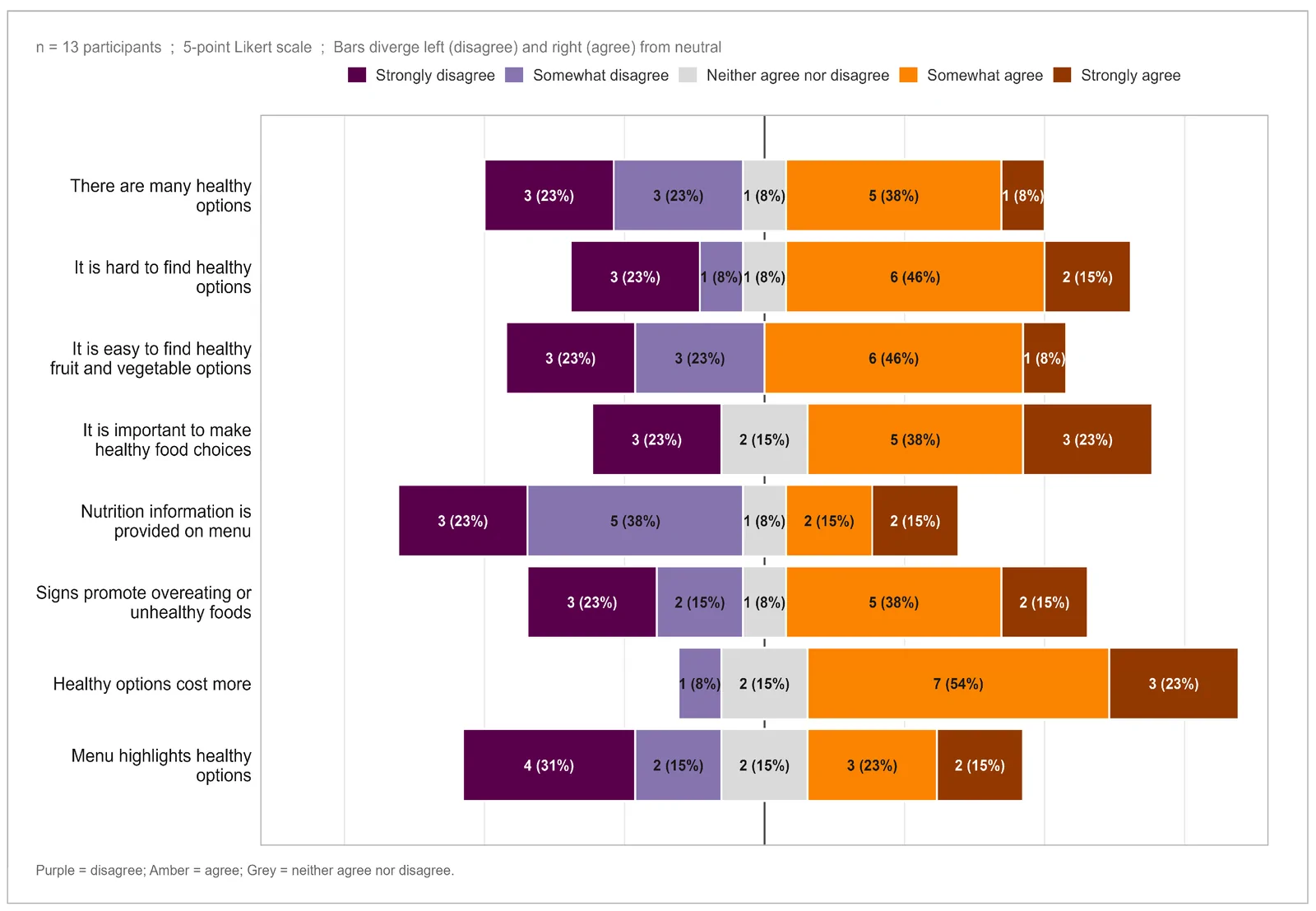
Youth’s perceptions of the restaurant environment based on the Perceived Nutrition Environment Measures Survey (NEMS-P).

Youth commonly characterized the food they obtained from fast-food restaurants and the drop-in center as unhealthy. Most participants reported in the survey that it was important to make healthy food choices, but they also reported it was hard to find healthy options at the restaurants where they ate most often (Figure 1). This perception was supported by observational quantitative data. Of the 589 main entrees available at fast-food and sit-down restaurants, only 27 met the NEMS definition of healthy. Notably, the 27 healthy entrees were spread across nine restaurants, meaning most restaurants had no healthy entrees available by the NEMS definition. Responses were mixed regarding participants’ perceptions about the availability of many healthy options and ease of finding healthy fruit and vegetable options at restaurants. On average, fast-food and sit-down restaurants scored 3.64 (sd = 3.97) out of a possible 21 or 30 (depending on the presence of a kid’s menu) on the NEMS-R (Table 2), suggesting the physical food environment at these sites discouraged health-promoting food choices and provided context to participants’ survey responses. As Jackson expressed: “I mean, we don’t sit there and think ahh, I want some fat-a** McDonald’s today. Or I want some Wendy’s.” At the drop-in center, additional descriptors of food included “expired” (multiple participants) and “leftovers” (Tyrell). Jelani expressed:

“Go out there and look in the ‘fridgerator’. If they bring anybody in here like whoever supposed to come check for code for food, bro, this place will be shut down. Everything in there is expired… I mean everybody eat it though, ‘cause they all, everybody obviously homeless and hungry… But I don’t think it’s healthy for something to be expired. It be healthy options, but it was like healthy last week. Like this week it’s expired.”

Participants reported a variety of self-led methods to cope with hunger, but the most reported strategy was “starving,” or fasting, described by Hallie as choosing not to eat “until I can find something to eat eventually.” Among those who reported fasting, participants cited a lack of desirable food as the most common justification. Some participants named religious beliefs, anxiety, weight control, and/or saving food for others as secondary reasons. Many participants engaged in other activities to distract from the feeling of hunger because, as Kyra stated, “when you don’t have nothing to eat it’s frustrating […] sleeping the entire day just to be okay, that’s something you do.” Other examples of hunger distraction activities included exercising, listening to music, and thinking about something other than food. Two participants shared that, after experiencing chronic food insecurity, they believed they did not have to eat as much to feel full. Still, participants acknowledged the biological necessity of food for survival, understanding that “you need to eat, so if you find something, just eat it” (Alicia). Hunger and the need for food drove many participants’ food choices, providing insight into why some participants ate food despite not liking or wanting it. Expanding on this notion, Tyrell stated:

“I don’t have a job right now, so it’s like, whatever, whatever [food] that comes to me, you feel me. Sometimes you gotta eat it to survive. So, I’m just on survival mode right now.”

### 3.3. Appetizing Food Is Unaffordable in Youth’s Environment

Price sub-scores for grocery and corner stores were 0.0 (sd = 2.0) and −1.0 (sd = 0.0), respectively, on a scale of −9.0 to 18.0 (Table 2). Lower scores indicated higher prices for lean meat and low-fat, low-sugar, whole-grain products compared to full-fat, full-sugar, and refined alternatives. Generally, food was available at stores, but product pricing decreased the affordability of healthy choices. Only nine of the 22 restaurants surveyed offered healthy entrees, seven of which had no cost difference between entrees that were healthy and unhealthy. In other words, only two of the restaurants had an upcharge for meals that met the NEMS definition of healthy. Yet, based on the interviews and surveys, participants overwhelmingly perceived healthy options to cost more at restaurants (Figure 1).

Findings were mixed regarding perceived importance of cost when purchasing food at restaurants or stores. Still, several participants described cost—both total cost and cost per serving—as a key barrier to obtaining desirable food. Some participants explained that they were more willing to spend money on foods that came in larger portions or to spend more money to buy their favorite foods; however, compromises were made to obtain these foods. Participants reported in their interviews that they often found free food and opted to buy relatively cheaper or cost-effective options. Riah, one of the few participants to report SNAP participation, explained their situation:

“What can you get with $67 [from SNAP]? And it’s not even like $67 a week, it’s $67 a month. So that’s why me and my sister have to go in on the groceries together because there’s not enough money. And then we also try to get like a lot of frozen foods opposed to fresh because we know it’ll last us longer, or the preservatives like they’ll help the food last. It doesn’t, it doesn’t really suffice. Or because we may not have enough money to get the foods that we want, we’ll end up getting junk foods because they’re cheaper. And then they don’t last, so, because you’re not, you’re never really full off junk food. At least I’m not. And it doesn’t feel good getting full off junk food either.”

According to participants, the drop-in center and other charitable organizations provided and acted as important resources when applying for SNAP benefits. However, most participants were not receiving SNAP benefits at the time of the study; only three participants indicated in their survey or interview responses that they were receiving SNAP benefits. Some participants explained in the interviews that they had received SNAP in the past but were not currently receiving benefits due to issues with the application/re-application process. For most participants, this meant not receiving email or mail correspondence until after the date of their SNAP appointment had passed.

### 3.4. Appetizing Food Is Inaccessible in Youth’s Environment

Participants discussed several transportation barriers to accessing desirable food. In both the surveys and interviews, walking and busing were reported as the most common transportation methods participants used to access food. Other transportation methods included driving a car, or getting a ride from others. No participants reported using a bike to access food. Three participants had their own car and reported driving their car as their sole method of transportation to access food; however, the cost of gas and upkeep were reported challenges. Participants explained that free bus passes were provided at the drop-in center, but there were often not enough for everyone. Some participants expressed personal safety concerns such as getting hit by a car or walking late at night. Witnessing or previously experiencing physical violence on the streets was reported as a cause of concern among some participants. Of note, these same participants emphasized their ability to physically defend themselves, stating that they learned to fight from their experiences growing up in the “hood”. Another concern raised by participants was the weather, specifically the rain, heat, and cold. Tyrell shared:

“I hate the cold weather. Who wants to walk in the cold? Makes my bones hurt. Like it’s terrible… I went outside, four days ago, to go to the store. I only made it like five steps before I turned around.”

Participants reported in their interviews that the shared kitchen facilities at the drop-in center further limited the accessibility of desirable food. Participants were aware they could use the kitchen at the drop-in center, including the appliances and utensils, but several participants explained they would not use it due to issues with sharing, concerns about the cleanliness of the kitchen, a lack of “the right appliances” and a dislike of cooking in front of others. Most common, however, were instances where youth witnessed or experienced having their food stolen or thrown out by other youth at the drop-in center. As Tyrell explained, “if you don’t get to [your food], somebody else will.”

Many youth discussed eating meals alone, and several participants expressed a reluctance to ask for help accessing food. When asked about accessing food through their social networks, youth’s responses ranged from mild hesitancy to a strong distaste. Participants who took offense at the idea of being a “food leech” (Jelani) stated they were “not that kind of person” (Andre) and “don’t use people” (Jackson & Alicia). Kyra explained, “I don’t like going to people’s houses ‘cause I don’t like asking for food and all that, so I mostly come to the [drop-in center].” Some of these youth, such as Jackson, resorted to stealing, panhandling, and dumpster diving to cope with their lack of food but were unwilling to ask for help from those in their social network. This shared rejection of exploiting others for food provides additional context to why youth felt appetizing food was inaccessible in their environment.

## 4. Discussion

This study described the perceived food environment and food choice among youth experiencing homelessness. Youth perceived food to be available in their environment, however available options were not affordable, accessible, or appetizing. Even when food was available, accessible, and affordable at the drop-in center or fast-food restaurants, youth experienced food insecurity and hunger because they did not perceive these foods as appetizing. In other words, youth needed but did not desire to eat these foods.

Based on the NEMS data, high quality, health-promoting foods such as fruit, vegetables, and lean meats were available at stores in the physical food environment. However, due to barriers such as perceived cost, lack of SNAP benefits, safe transportation, and personal food preparation and storage facilities, youth reported eating at the drop-in center and restaurants more often, although these were, respectively, perceived to have or actually had (based on observational data) lower availability of healthy food. The identification of the drop-in center as a major food source for YEH in this study is consistent with previous studies [4,5]. While the NEMS observational measures precluded our ability to assess food availability within the drop-in center in the current study, previous research conducted at the same site found that dry cereal, vegetables, and meat or non-dairy protein foods were the most commonly available items, whereas candy, dairy products, and frozen desserts were the least available [32]. The current findings provide context to this prior research, as youth explained that the available “healthy” foods were often deemed undesirable for consumption due to past expiration dates or being perceived as leftovers. The perception that expired foods are unsafe is widespread and not unique to YEH. Studies have shown that this belief contributes to increased consumer food waste, especially among young adults and economically vulnerable populations [33]. Our findings therefore highlight an opportunity to integrate nutrition and food safety education into drop-in center services to address both food and nutrition insecurity among YEH utilizing such services [34]. Such education may strengthen youths’ ability to identify, select, and prepare available foods in ways that enhance their perceived desirability. Nonetheless, it is important to note that studies of charitable meal programs in both the U.S. and Canada have found that average shelter meals may not provide adequate nutrients to meet recommended daily intakes [11,35]. In the U.S. specifically, a nutrient analysis of 22 meals from six different free sites showed that the meals provided high amounts of fat and salt but were concurrently deficient in fiber, potassium, calcium, and vitamins A and E [35]. Previous research suggests that access to free, health-promoting foods is inconsistent for those experiencing homelessness and likely depends on the food and monetary donations received by charitable organizations such as drop-in centers [35,36]. This further suggests that food access challenges among youth experiencing both homelessness and food insecurity may relate more to food quality than quantity. It also underscores the need for stakeholders to dedicate more resources to ensuring the consistent provision of high-quality foods to charitable organizations serving YEH. In the present study, 95% of restaurant meals did not meet the NEMS definition of healthy because they were too high in saturated fat and/or calories. This correlated with youths’ characterization of restaurant foods as unhealthy. The low health-promoting value of these foods were reflected in dietary intake data showing that 39–50% of total calorie intake for YEH was from fats and oils, condiments, alcohol, sugar sweetened beverages, and prepared dishes high in sugar, fat, and/or salt [37]. Similarly, inadequate nutrients intake, especially among female YEH, have been reported in prior research [16,17].

Our findings also show a need for improved enrollment in SNAP. Safety net programs such as SNAP are intended to alleviate food insecurity by providing monetary assistance; however, lengthy and bureaucratic enrollment procedures can prevent utilization by populations with the greatest need [38]. Difficulties related to paperwork, program staff, time constraints and competing priorities have been identified as barriers to SNAP participation among families experiencing homelessness. Similarly, YEH have reported challenges with the SNAP application and recertification process as well as being denied SNAP benefits when their parents did not remove them from their household SNAP applications [12,39]. Consistent with prior research, our findings show that study participants in the present study were not receiving SNAP benefits due, in part, to poor, untimely communication from the SNAP program. Unique to this study, however, is the very low rate of SNAP enrollment. Whereas prior studies had food assistance enrollments of 59% of focus groups participants, 75% of survey respondents, and 70% of YEH, [12,39], only three participants (21%) in the current study reported receiving benefits. This low participation rate may plausibly explain why youth relied so heavily on the drop-in center and infrequently shopped at stores. In other words, the drop-in center may have served as a more economically accessible and reliable source of food assistance, given the barriers to SNAP participation reported by YEH. In that sense, geographic differences in the availability of local food assistance resources, as well as variations in factors such as program policies, service hours, and accessibility, may have contributed to the lower SNAP participation observed in this study relative to the only prior study among YEH [12]. Additional research to better understand the factors influencing SNAP participation among YEH across different settings would be helpful.

Still, though the drop-in center and fast-food restaurants removed barriers to food availability, affordability, and accessibility, this did not resolve the experience of food insecurity. All youth in this study were experiencing food insecurity, with many experiencing severe food insecurity. For many youth, fasting was preferable to consuming foods they found unappetizing, which may have contributed to the high prevalence of food insecurity observed in this study. Although food may be physically available and accessible within youths’ environments, hunger and food insecurity can persist when available foods are perceived as undesirable or when food acquisition methods are viewed as unacceptable. Evidence supports the fact that youth are reluctant to seek assistance from members of their social networks, even in relation to food, and therefore rely on alternative coping strategies. The self-led strategies youth employ to adapt, cope, and survive are well documented in prior research on youth experiencing homelessness and food insecurity [4,12,15,40,41,42]. These findings suggest that food insecurity among YEH extends beyond the mere availability of food and is shaped by how youth perceive and engage with available food options.

In a prior study on youth experiencing homelessness, participants most frequently identified income, stable housing, and access to kitchen facilities as what would most improve their access to food. Our findings, consistent with prior research, also suggest that improving both access to and the quality of food-related services provided through safety-net programs could enhance food access among YEH [14]. Safety-net programs such as drop-in centers and SNAP are essential components of the food environment for many YEH and play an important role in mitigating food insecurity. However, these programs represent only an initial step and require continued improvement to enhance their quality, accessibility, and effectiveness. Potential interventional strategies towards optimization could include increasing resources to improve the quality and accessibility of foods provided by charitable organizations, integrating advocacy and support services to assist youth in navigating available food resources, and providing tailored education that builds youths’ self-efficacy in nutrition, food safety, and food resource management while accounting for the unique needs of YEH and challenges associated with homelessness. Nutrition education may be especially beneficial in helping YEH make healthier food choices within fast-food settings, which emerged as a component of their food environment. Nevertheless, sustainable improvements in the food environment of YEH will require multidisciplinary approaches that address the underlying drivers of both housing and food insecurity and will depend on coordinated efforts among stakeholders across the housing, employment, nutrition, health care sectors, etc. [15].

This study had several strengths. Most notably, the study was conducted within a hard-to-reach population that requires rapport building. Additionally, using a mixed-methods approach allowed for deeper analyses and understanding of the phenomena studied. Finally, the research team consisted of individuals who are diverse in age, ethnicity, educational attainment, and geographical location. This is presumed to strengthen the study, as it increases the number of experiences, perspectives, and interpretations available to researchers during analyses.

This study also has several limitations. First, the NEMS-P has not been validated for use among populations experiencing housing instability, which may limit its applicability to YEH, especially with regards to the assessment of the home food environment. Nevertheless, the mixed-method design allowed for the integration of qualitative data to examine youth’s perspectives of their “home” food environment beyond the dimensions covered by the NEMS-P. Similarly, the NEMS observational measures were limited to fast-food or sit-down restaurants, grocery stores and corner stores. As a result, the availability of food at other sources identified through the interviews, including the drop-in center, could not be assessed systematically using quantitative methods. However, prior research conducted within the same drop-in center provided additional contextual insight into the role of this setting as an important food source for YEH. Additionally, since the study team honored participants’ preferences regarding the sequence of completing research activities, it is possible that the order in which these activities were completed may have influenced participants’ responses and the interviewer’s ability to formulate follow-up questions. Finally, this study included a convenience sample of youth experiencing homelessness who accessed services at a single drop-in center in one city. As such, the findings may not be generalizable to YEH living in other settings, experiencing different circumstances, or having access to different resources. Nonetheless, this study contributes to the limited literature on food environments among YEH as it centers on youths’ perspectives and helps identify potential targets for intervention.

## 5. Conclusions

This study examined how youth experiencing homelessness perceive their food environment and how these perceptions influence their food choices. Findings showed that food was available to youth from various sources, however, some of these sources were perceived as unaffordable or otherwise inaccessible. Conversely, foods obtained from accessible sources, such as a drop-in center and fast-food restaurants, were perceived as unappetizing or undesirable. These perceptions may partially explain the paradox of food insecurity reported by participants despite the availability of food in their environment. Therefore, food access among YEH depends not only on availability but also on the affordability, accessibility, quality, and desirability of available foods. Interventions are needed to address these food environment barriers to include providing tailored resources and education to support safe, nutritious, and acceptable food choices among YEH.

## Figures and Tables

**Table 1:** Participant demographic and homeless experience characteristics.

Characteristic	*n* (%)
Age, years	
18–20	6 (43)
21–24	8 (57)
Sex	
Male	8 (57)
Female	6 (43)
Race	
Black, African American	9 (64)
Native American, Alaskan Native	1 (7)
White	2 (14)
Other	2 (14)
Ethnicity	
Hispanic	1 (7)
Non-Hispanic	13 (93)
Education	
Grade ≤ 9	3 (21)
Grade 10–11	3 (21)
Grade ≥ 12	8 (57)
Employment Status	
Part-time (fewer than 40 h/wk)	4 (28)
Unemployed/Medical disability	5 (36)
Student	5 (36)
Monthly Income, USD ^a^	
0–1000	7 (58)
1000–2000	2 (17)
2000+	3 (25)
Receiving SNAP benefits	
Yes	3 (21)
No	11 (79)
Age at First Homeless, years	
1–12	2 (14)
12–18	5 (36)
18+	7 (50)
Homeless Episodes ^b^ m (sd)	6.23 (7.58)
Current Homeless Duration, months; m (sd)	8.93 (10.15)
Subsistence Strategies ^c^	
Full- or part-time job	9 (64)
Day labor/Seasonal work	5 (36)
Pawning	1 (7)
Panhandling/spanging	1 (7)
Selling blood/plasma	2 (14)
Selling drugs	2 (14)
Survival sex	1 (7)
Stealing	3 (21)
Primary Housing ^c^	
Friend or family member’s house	2 (14)
Shelter or mission (including drop-in center)	10 (71)
Car, abandoned building, or public indoor space	6 (43)
Outside location	5 (36)

^a^ *n* = 12; two participants declined to disclose income level; ^b^ *n* = 13; one participant did not report; ^c^ do not sum to 100% because youth could mark multiple answers; SNAP: Supplemental Nutrition Assistance Program.

**Table 2:** Nutrition Environment Measures Survey (NEMS) scores for restaurants, stores, and corner Stores.

Store Type	Availability Sub-score (range 0 to 15) *	Facilitators Sub-score (range 0 to 8) *	Barriers Sub-score (range −5 to 0) *	Total Score (range −5 to 23) *
† Fast food/Restaurant (R), *n* = 22	3.14 (2.68)	2.45 (1.18)	−1.95 (1.13)	3.64 (3.97)
	Availability Sub-score (range 0 to 30) *	Price Sub-score (range −9 to 18) *	Quality Sub-score (range 0 to 6) *	Total Score (range −9 to 54) *
† Store (S), *n* = 3	25.67 (1.15)	0.00 (2.00)	6.00 (0.00)	31.67 (3.06)
	Availability Sub-score (range 0 to 37) *	Price Sub-score (range −9 to 18) *	Quality Sub-score (range 0 to 6) *	Total Score (range −9 to 61) *
† Corner Store (CS), *n* = 1	16.00	−1.00	4.00	19.00

* Mean (SD); † Nutrition Environment Measures Survey (NEMS) tools for Restaurants (NEMS-R), Stores (NEMS-S), and Corner Stores (NEMS-CS) were scored using standardized scoring procedures that produce an overall score based on food availability, pricing, quality, facilitators, and/or barriers sub-scores. Higher total scores indicate greater availability and accessibility of healthy food at sites.

## Data Availability

The qualitative data are not publicly available because sharing them could compromise participant confidentiality. De-identified raw quantitative data are available from the corresponding author upon reasonable request.

## Abbreviations

The following abbreviations are used in this manuscript:

NEMS	Nutrition Environment Measures Survey
NEMS-P	Perceived Nutrition Environment Measures Survey
NEMS-R	Nutrition Environment Measures Survey for Restaurants
NEMS-S	Nutrition Environment Measures Survey for Stores
NEMS-CS	Nutrition Environment Measures Survey for Corner Stores
SNAP	Supplemental Nutrition Assistance Program
HFSSM	Household Food Security Survey Module

## Appendix A. Interview Guide

Priming/Rapport Question:

1. If you had to eat one meal for the rest of your life, what would it be?
2. What kinds of foods do you most enjoy eating?

Person-Centered Food Environment

3. What foods and drinks did you consume today?
  - Are these the types of foods you eat on a typical day?
  - Are these the types of beverages you drink on a typical day?
  - What factors did you consider when choosing what to eat and drink?
  - What barriers did you face, if any, in choosing foods that are considered “healthy,” such as fruit, vegetables, lean meats, and low-fat dairy products?
  - How much confidence did you have in your ability to cook and prepare the food?
  - To what extent did you enjoy or not enjoy cooking and food preparation?
  - How do you think what you ate and drank today impacts your health?
  - What do you think of the quality of food provided at shelters and drop-in centers?
4. How soon after waking up today did you begin to think about finding something to eat or drink?
  - What emotions did you feel when you thought about food?
  - [only ask if respondent indicates negative emotions in answering preceding questions] How did you cope with these negative emotions when they arose?
  - How did finding something to eat or drink impact how you planned your day?
  - How did finding food affect what you were able to accomplish in the day?

Social Food Environment:

5. How many people would you identify as being in your social network?
  - What is your relationship to each of these people?
  - How did you meet these people?
  - Please describe the last time, if any, you contacted or hung out with someone because they had food or could help you access food.
6. Considering the people in your social network, when was the last time each of them provided food for you or helped you access food?
  - For those who gave you food, what kinds of food did they provide?
  - The last time you hung out with those in your social network, what foods and drinks did you share?
  - Was this different than the foods you would have eaten if you had been by yourself?
  - Please describe to me a moment, if any, that you felt judged for the types of food you ate or how much you ate.
  - [skip if respondent does not identify having a pet on their sociodemographic survey] I see from your survey that you have a pet! Please describe a time, if any, when feeding your pet impacted what you ate.
  - What food, resource, or skill have you been given by those in your social network that has been most helpful for you?
7. In what social setting did you consume most of your food today, including the physical location and the people who were there?
  - Did you feel pressure in this setting to behave a certain way?
  - What formal rules does this setting have, if any, about food and/or meals?
  - What informal, implied rules does this setting have, if any, surrounding food and/or meals?
  - It’s common to get in disagreements with another individual or individuals at the places where we socialize. Would you please describe the worst experience you have had with another individual in the places where you eat food?
  - Could you please describe the worst experience you have had with another individual, such as security officers, store employees, or other customers, at the places where you purchase food?

Physical Food Environment

8. Where have you gone in the last month to access food and water?
  - How often did you go to each of these places to access food or water?
  - What type of transportation did you use to reach these places?
  - How close were these places to where you stay at night?
  - How long did it take you to reach these places?
  - What concerns did you have, if any, about safety when you were at or traveling to these places?
  - How did the weather, if at all, impact your ability to access these places?
9. I see from your survey that you [do/do not] currently receive Supplemental Nutrition Assistance Program, or SNAP, benefits.
  If YES: Could you please describe your experience applying for SNAP?
  - If you are comfortable sharing, would you mind telling me how much money you received last month in SNAP benefits?
  - How long did the money last?
  - What foods did you purchase with these SNAP benefits?
  - To what extent did your ability to use SNAP at a store impact whether you shopped there?
  - What other national or local assistance programs did you use, if any, to supplement the SNAP benefits?
  - What other strategies have you used in the past month to obtain food?
  If NO: Have you ever received SNAP in the past?
  - (i) If YES: Could you please describe for me the time when you decided to no longer utilize SNAP?
    (ii) If NO: Why have you chosen not to receive SNAP?
  - What other national or local assistance programs do you use, if any, to access food?
  - What other strategies have you used in the past month to obtain food?

10. What food preparation and storage facilities did you have access to at least three days of the last week?
  - How do these facilities differ from the facilities available in the place where you sleep at night?
  - In facilities that are for communal use, could you please describe the last time, if any, that conflict occurred over sharing appliances?
  - What appliance do you currently lack that you feel would most improve your access to food?

General Question

11. Considering the different physical, social, and personal aspects we’ve discussed today, what top three things would improve your current diet?

Closing

12. Was there anything you expected to talk about today that we did not discuss?
13. Is there anything more you would like to add before we finish?
